# Human deprivation amblyopia: treatment insights from animal models

**DOI:** 10.3389/fnins.2023.1249466

**Published:** 2023-09-19

**Authors:** Kevin R. Duffy, Mark F. Bear, Nimesh B. Patel, Vallabh E. Das, Lawrence Tychsen

**Affiliations:** ^1^Department of Psychology and Neuroscience, Dalhousie University, Halifax, NS, Canada; ^2^Picower Institute for Learning and Memory, Department of Brain and Cognitive Sciences, Massachusetts Institute of Technology, Cambridge, MA, United States; ^3^College of Optometry, University of Houston, Houston, TX, United States; ^4^Department of Ophthalmology and Visual Sciences, Washington University School of Medicine, St. Louis, MO, United States

**Keywords:** amblyopia, monocular deprivation, neural plasticity (NP), animal models, neuropathology, amblyopia therapies, recovery

## Abstract

Amblyopia is a common visual impairment that develops during the early years of postnatal life. It emerges as a sequela to eye misalignment, an imbalanced refractive state, or obstruction to form vision. All of these conditions prevent normal vision and derail the typical development of neural connections within the visual system. Among the subtypes of amblyopia, the most debilitating and recalcitrant to treatment is deprivation amblyopia. Nevertheless, human studies focused on advancing the standard of care for amblyopia have largely avoided recruitment of patients with this rare but severe impairment subtype. In this review, we delineate characteristics of deprivation amblyopia and underscore the critical need for new and more effective therapy. Animal models offer a unique opportunity to address this unmet need by enabling the development of unconventional and potent amblyopia therapies that cannot be pioneered in humans. Insights derived from studies using animal models are discussed as potential therapeutic innovations for the remediation of deprivation amblyopia. Retinal inactivation is highlighted as an emerging therapy that exhibits efficacy against the effects of monocular deprivation at ages when conventional therapy is ineffective, and recovery occurs without apparent detriment to the treated eye.

## Amblyopia and its subtypes

Normal development of the mammalian visual system begins with a prenatal sequence of patterned gene expression that interacts with spontaneous electrical activity to produce rudimentary neural circuits (Kuljis and Rakic, 1990). Although the primate visual system exhibits some mature physiological and anatomical properties at birth (Wiesel and Hubel, 1974; Horton and Hocking, 1996), its development at this stage is insufficient to support adult visual perception (Teller and Boothe, 1962). Human infants have limited visual acuity (Brown and Yamamoto, 1986) and are unable to assemble visual details into a whole percept (Cohen and Younger, 1984). Visual experience early in postnatal life directs the maturation of neural circuitry to optimize function of the visual system and produce clear binocular vision. The important synergy between visual experience and neural development is facilitated by a high capacity for neural plasticity that occurs naturally only early in postnatal life, during the so-called critical period (LeVay et al., 1980; Olson and Freeman, 1980; Hensch, 2005; Mitchell and Maurer, 2022). Although this is a formative stage in normal neural development, it also represents a time of vulnerability wherein impressionable neural circuits can be misguided by conditions that interfere with the piloting influence of normal concordant vision. Amblyopia is a visual impairment caused by aberrant neural development in the primary visual pathway resulting from early abnormal visual experience.

Amblyopia is the leading cause of monocular vision loss in the U.S., affecting approximately 2.2 million children (Friedman et al., 2009), and is the most common cause of monocular visual impairment in adults (Grant and Moseley, 2011). It develops as a sequela to misaligned eyes (strabismus), an imbalanced refractive state (anisometropia), or an obstruction to form vision (deprivation). Each of these conditions blocks binocular concordance and derails the typical development of neural connections, seeding persistent abnormalities within brain regions that assemble visual perception. Dysfunctions that result from this cascade of events are many, but foremost among them is a pronounced reduction in spatial acuity in the affected eye (Ellemberg et al., 2000), impairments to fine motor skill and coordination (Kelly et al., 2019), and a reduced or absent capacity for stereoscopic vision (Wallace et al., 2011).

Strabismic and anisometropic amblyopia are the most common subtypes of the disorder (Pediatric Eye Disease Investigator Group, 2002), with deprivation amblyopia being comparatively rare and representing only about 3–4% of amblyopia cases (Flynn and Cassady, 1978; Attebo et al., 1998). Although less common, deprivation amblyopia is by far the most severe subtype, and is the least responsive to conventional treatment. It derives from early onset monocular deprivation (MD) or binocular deprivation. While our focus will be on characteristics of MD, a comparison of the effects of monocular and binocular deprivation has been reviewed elsewhere (Maurer, 2017). The majority of published cases of deprivation amblyopia are due to cataract, which causes the disorder by obstructing the focus of images on the retina. Whereas anisometropic amblyopia started in infancy can take months to develop (Smith et al., 1985), 2 weeks of MD imposed at the same age is sufficient to produce a near complete loss of spatial vision in monkeys (Harwerth et al., 1981). Suppression of the amblyopic eye is stronger in children with deprivation amblyopia compared to those with anisometropic or strabismic amblyopia (Hamm et al., 2017). Treatment outcomes for amblyopia in infants with congenital unilateral cataract before the 1980’s were dismal. Recovery attempts were considered pointless (von Noorden and Maumenee, 1967), and some early published reports even recommended against therapy (Costenbader and Albert, 1957; Ryan and Maumenee, 1977).

**Figure 1:**
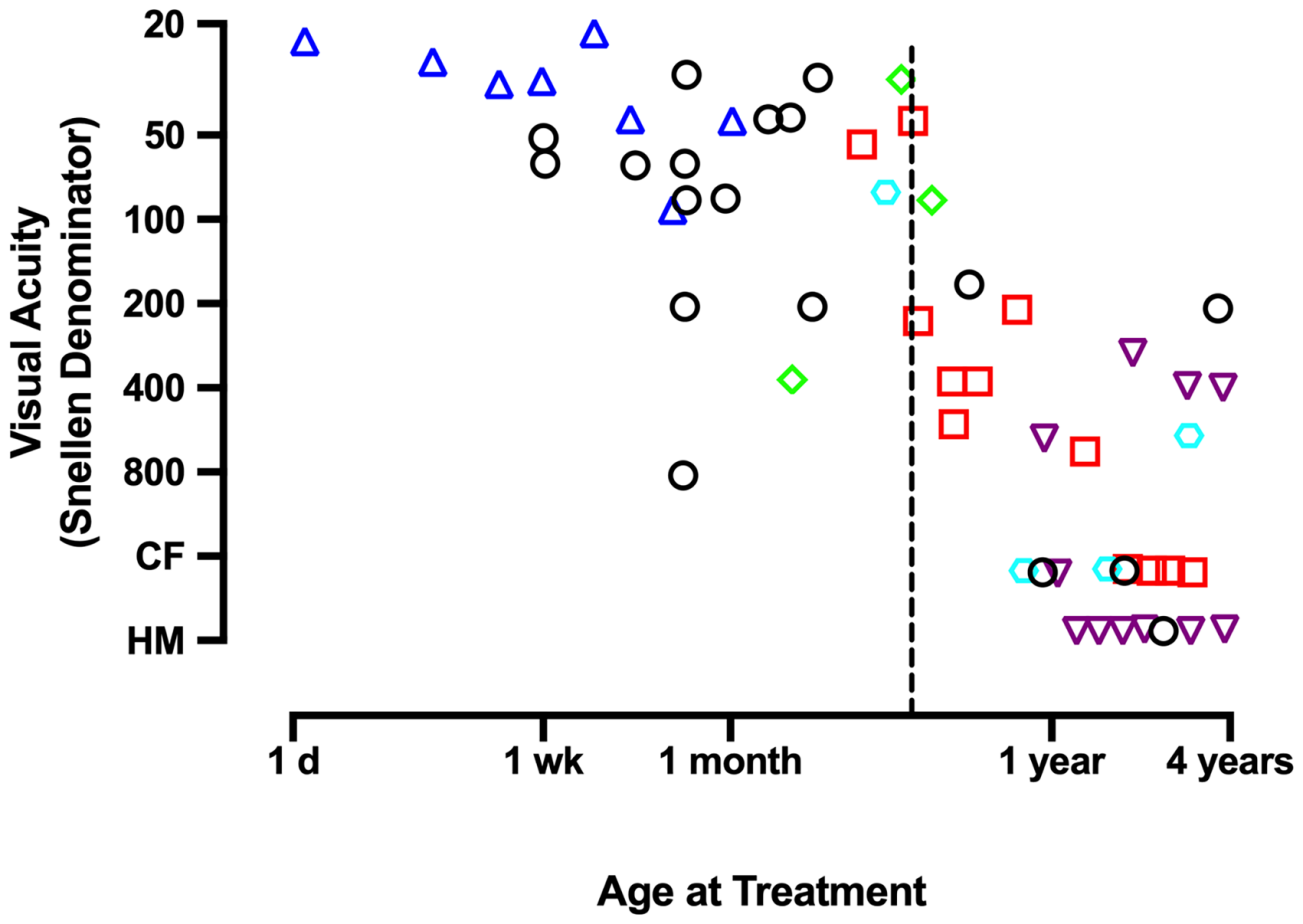
This graph plots the Snellen acuity achieved by the amblyopic eye as a function of the age at which a unilateral congenital cataract was removed and occlusion treatment began. Data demonstrate that, unlike other forms of amblyopia, effective treatment of deprivation amblyopia adheres to a short critical period in which, to promote optimal recovery from congenital MD, therapy must begin before the age of about 4 months (dashed vertical line). Therapy initiated beyond 4 months of age is associated with poor recovery outcomes. Graph displays results that were compiled by Birch and Stager (1988). Data originate from Beller et al., 1981 (triangles); Lewis et al., 1986 (squares); Pratt-Johnson and Tillson, 1981 (diamonds); Helveston et al., 1980 (hexagon); Awaya et al., 1979 (inverted triangles); Birch and Stager, 1988 (circles). *CF* indicates ability to count fingers; HM indicates the perception of hand movement.

Insights that emerged from the discovery of a critical period in animal studies measuring the effects of MD (Wiesel and Hubel, 1963a,b; Hubel et al., 1970) motivated new attempts to examine recovery potential in humans treated for unilateral congenital cataract very early in life. These studies revealed that recovery was possible under strict conditions that included removal of the cataract shortly after birth, provision of corrective contact lenses, and compliance with occlusion therapy (Beller et al., 1981; Birch et al., 1986; Birch and Stager, 1988). Under these ideal circumstances, treated infants under 4 months of age were able to achieve visual acuity that was better than previously thought possible, but was sometimes still within the range of low vision and well short of normal acuity (Figure 1). Infants treated beyond 4 months of age suffered far worse outcomes that range from about 20/160 visual acuity to perception of hand motion (Birch et al., 1986; Birch and Stager, 1988). Even after an excellent standard of care delivered in the prospective National Eye Institute Infant Aphakia Treatment Study (NCT00212134), the average visual acuity achieved in children with MD was 20/160. Therefore, the common understanding that amblyopia can be successfully treated up to about 7 years of age (Holmes et al., 2011; Holmes and Levi, 2018) does not apply to amblyopia caused by MD. Instead, the disorder appears to express an early and ephemeral response to treatment and can improve only under strict therapeutic conditions. For these reasons it has been recommended that surgery, optical correction, and occlusion therapy be implemented before 6 weeks of age to avoid debilitating impairment (Birch and Stager, 1996). Although better outcomes are achieved as the amount of patching increases, good outcomes occur only when there has been both early treatment and extensive patching (Birch et al., 1993; Lewis et al., 1995; Drews-Botsch et al., 2012). These characteristics underscore the severity of MD and distinguish it from the other amblyopia subtypes.

Traditional monocular therapies have been employed for centuries and remain the gold-standard treatment for all types of amblyopia (Levi, 2020), but they are plagued by adherence issues (Stewart et al., 2003; Holmes and Levi, 2018), recurrence of amblyopia after treatment (Levartovsky et al., 1995; Jia et al., 2022), and negligible efficacy at older ages (Birch and Stager, 1988; Wallace et al., 2018). The social stigma associated with patching can raise stress and anxiety, and adversely impact the child–parent relationship (Awan et al., 2005; Loudon et al., 2009; Birch et al., 2019a). Moreover, monocular approaches like patching do not restore the loss of stereoscopic vision (Wallace et al., 2011), which is the most common binocular deficit of amblyopia (Webber and Wood, 2005). Therefore, in recent years, investigation into more effective treatments for human amblyopia has shifted focus away from traditional patching or penalizing the dominant eye to stimulate recovery (Koo et al., 2017). Emerging therapies investigated in humans aim to overcome the issues with monocular therapy through engaging and binocular-based approaches (reviewed in Levi, 2020; Bui Quoc et al., 2023). Some of the additional motivation to pursue binocular treatments has been recognition that the fellow eye is not “normal” and that there are significant binocular deficits that cannot be explained by monocular loss in acuity alone (Murphy et al., 2015; Meier and Giaschi, 2017; Birch et al., 2019c). Binocular treatments are designed to not only restore spatial acuity for the impaired eye but also to promote recovery of binocularity and stereopsis. Recovery of stereovision is an achievement that typically eludes traditional monocular therapies. Notwithstanding the potential benefits that these innovative amblyopia treatments may offer to patients, investigation of their efficacy has focused almost entirely on strabismic and anisometropic amblyopia (Hamm et al., 2017). The exclusion of patients with MD from these studies, as well as from National Eye Institute-sponsored Amblyopia Treatment Studies of the Pediatric Eye Disease Investigator Group (Chen and Cotter, 2016), derives from the obstinance and poor prognosis for recovery associated with this subtype of amblyopia.

Given the severity and intractable characteristics of deprivation amblyopia, and the paucity of human studies focused on its remediation, development of a novel treatment that provides superior recovery, and at older ages, would be clinically transformative. Broadening the age limits of successful treatment would alone represent a major discovery (Levi, 2020). A treatment innovation capable of promoting recovery from the most recalcitrant form of amblyopia may also have superior efficacy for the remediation of the less severe amblyopia subtypes: anisometropic and strabismic.

## Animal models: strengths and limitations

Decades of research into the rootedness of amblyopia using animal models has overwhelmingly employed closing the lids of one eye to produce form MD. The ease of its production alongside the large, rapid, and consistent effects that it yields have made lid closure the most prolific method to produce amblyopia in animal models. This has occurred despite the fact that MD is the rarest and most recalcitrant form of amblyopia in humans (Flynn and Cassady, 1978; Attebo et al., 1998). Anisometropic and strabismic amblyopia are both effectively modeled in cats and monkeys (Kiorpes et al., 1998), and can occur naturally in these species (Berman and Payne, 1983; von Grünau and Rauschecker, 1983; Horton et al., 1997; Tychsen et al., 2004). Nevertheless, there are a dwindling number of animal studies that induce these more common forms of amblyopia to investigate etiology and recovery. Increasing use of anisometropia and strabismus to model amblyopia would enable better correspondence with the diversity of amblyopia subtypes in the human population and would also produce better alignment between animal work and the majority of human studies that examine only these conditions.

Although the preponderance of MD studies in animal models may present challenges for expeditious clinical translation across amblyopia subtypes, insights gained from studies using MD continue to be at the vanguard of our knowledge about the regulators of neural plasticity as well as the organization and development of the primary visual pathway. The robust effects of MD in animals facilitates exploration of unconventional approaches to therapy that cannot be developed in human subjects. Further, effective treatments developed and tested in animal models using MD are likely to provide relief from all types of amblyopia for at least two reasons. First, strabismic, anisometropic and MD amblyopia all respond to the same treatments in the clinic (albeit with limitations indicated above). Second, therapy effective against the deepest and most obstinate form of amblyopia should also be efficacious for the more treatable subtypes. Using MD to model amblyopia sets the bar high; indeed, in monkeys the induced impairment can be more severe than that observed in humans (Kiorpes, 2019).

In recent years, the prolific use of rodents (particularly mice) in visual neuroscience has made them a standard model for investigating mechanisms of neural plasticity that underlie the emergence of amblyopia and that enable its recovery (Heynen et al., 2003; Morishita et al., 2010; Kaneko and Stryker, 2023). The mouse has a poorly differentiated visual system. Although the power of mouse genetics is undeniable, the primitive organization of the mouse visual system gives rise to limitations on what can be deduced in humans. Many characteristics that are the exclusive domain of cortex in higher mammals appear to be residual in rodents. This may offer interesting insight into the evolution of the visual system, but it does complicate direct translation of knowledge gained from rodent models to other species with more highly differentiated visual systems. The exorbitant expense, long gestational times, protracted postnatal development, and small litter sizes make mechanistic studies difficult to perform on cats and monkeys. Rodent studies have been paramount in delineating important characteristics of visual system plasticity including the discovery that the mature mammalian brain retains considerable capacity for neural plasticity beyond what was previously thought (Pizzorusso et al., 2002; Sawtell et al., 2003; He et al., 2007; Bavelier et al., 2010; Morishita et al., 2010; Fong et al., 2021). These findings have motivated investigation of the limits of plasticity and recovery from anisometropic and strabismic amblyopia in humans. Although some promising results have been observed (Sharif et al., 2019; Wu et al., 2023), translating these treatment innovations has not been straightforward (Repka et al., 2015; Sofi et al., 2016; Chung et al., 2017; Lagas et al., 2019).

**Figure 2:**
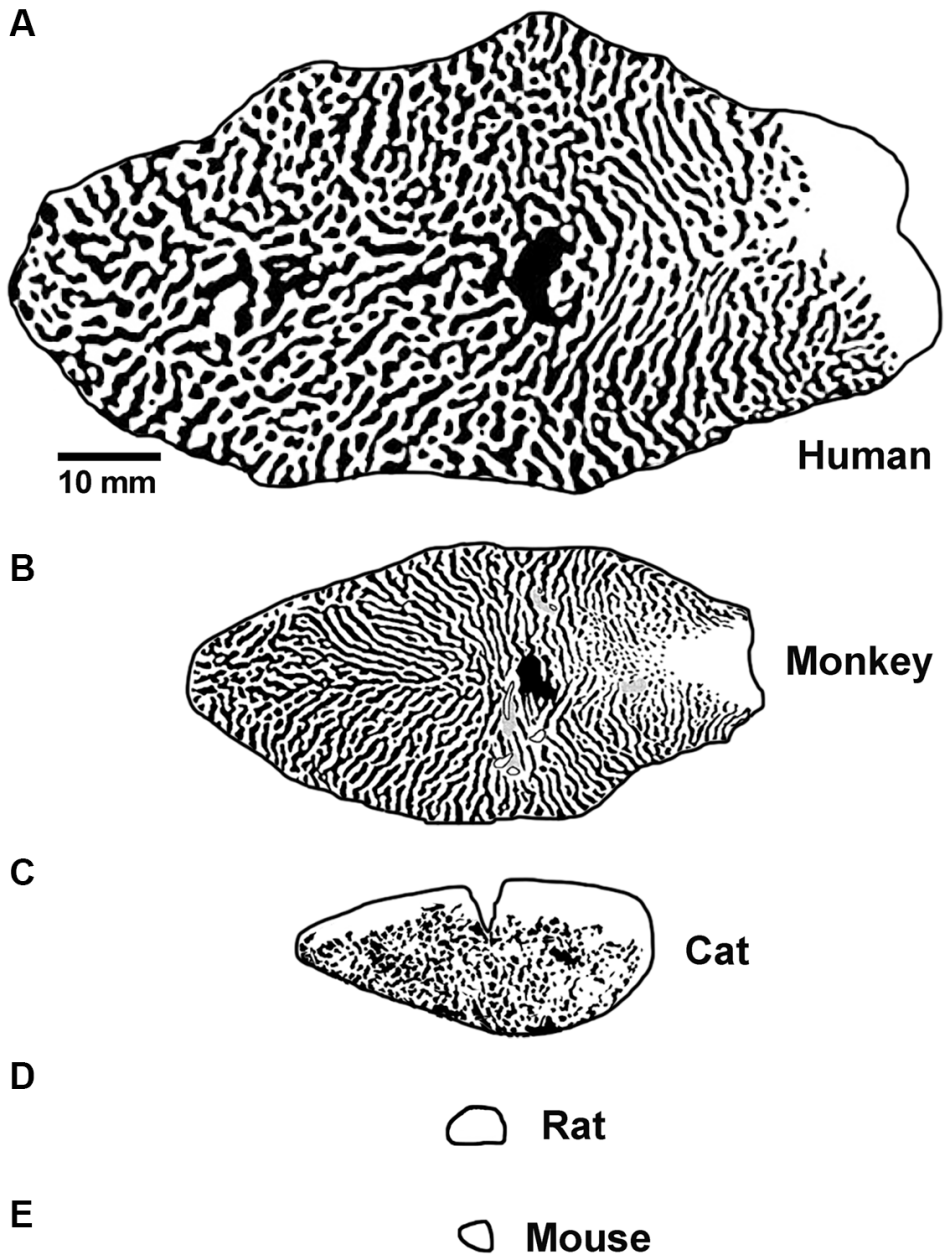
Graphical representation of the surface area and tangential ocular dominance organization of V1 for human **(A)**, macaque monkey **(B)**, cat **(C)**, rat **(D)**, and mouse **(E)**. Note the progressive decrease in overall surface area of V1 moving from human to mouse, and also the difference in organization observed across species. Scale bar is 10 mm. Images and organizational details of V1 originate from Adams et al. (2007) (human), Horton and Hocking (1996) (macaque monkey), Anderson et al. (1988) (cat), Duffy et al. (1998), Laing et al. (2015) (rat), and Airey et al. (2006) (mouse).

Differences between the visual systems of rodents and humans may represent a formidable obstacle to the smooth transition from bench to bedside. Among the common animal models for amblyopia, visual spatial acuity is highest in monkeys (30 cycles / degree; Kiorpes, 1992), followed by cats (8–10 cycles / degree; Giffin and Mitchell, 1978; Murphy et al., 2015), then rats (1 cycle / degree; Prusky et al., 2000) and finally mice (0.5 cycles / degree; Prusky and Douglas, 2003). Monkeys and cats have forward-facing eyes and a large binocular zone in visual space. Rats and mice have lateral facing eyes and a small binocular zone with poor stereopsis (Baroncelli et al., 2013; Boone et al., 2021). The structure and function of the primary visual pathway in rats and mice is likewise substantially different. These burrowing rodents do not have a laminated lateral geniculate nucleus (LGN) with eye-specific layers, and unlike primates and carnivores, sensory receptive fields can exhibit both orientation selectivity and binocular responses (Suresh et al., 2016). Moreover, the responses of binocular neurons in murine LGN are modified by MD. This plasticity is not a passive reflection of feedback from V1 and appears to result from changes in the retinogeniculate synapses (Jaepel et al., 2017; Sommeijer et al., 2017; Huh et al., 2020). In primary visual cortex (V1), rodents do not have a human-like ocular dominance organization. Instead of stripes or patches of ocular dominance as is observed in human, monkey and cat, eye-specific geniculocortical inputs to mouse V1 are mixed within a single binocular zone dominated by input from the contralateral eye (Coleman et al., 2009). In rats, inputs to V1 exhibit regions of aggregated eye-specific input within the binocular zone, but these domains appear distinct from those observed in higher species (Laing et al., 2015). In comparison to humans, the overall amount of cortical territory taken up by V1 is markedly smaller for all commonly studied model species, including monkeys (Figure 2). The surface area of macaque monkey V1 is about 40% of human V1, and for cats this is even smaller at 15%. However, these size differences are dwarfed by those observed for rat and mouse V1, which are about 0.5% the area of human. The difference in potential computation capacity of V1, alongside their very poor visual ability, has fueled the view that rodents are suboptimal models for studies of human vision (Baker, 2013). Regarding studies of amblyopia, a notable conspicuous limitation to using rodents is that only deprivation amblyopia has been successfully modeled. The apparent inability for rodents to express the two most common subtypes of human amblyopia, namely anisometropic and strabismic amblyopia, presumably derives from their dissimilarities compared to humans, which include those already mentioned as well as others reviewed elsewhere (Espinosa and Stryker, 2012; Baker, 2013; Seabrook et al., 2017; Mitchell and Sengpiel, 2018).

A suggested approach to mitigate failures in extrapolating from animals to humans is a “two-species rule”: a result in one species should be confirmed in at least one other, ideally a non-human primate (Mitchell and Sengpiel, 2018; Levi, 2020). As a model of human amblyopia, non-human primates offer significant advantages. Monkeys are similar to humans in possessing a fovea, have excellent spatial vision, stereopsis, analogous visual cortical pathways, and a response to amblyogenic rearing that closely mimics the human condition (see reviews from Kiorpes, 2019; Tychsen, 2020). Parlaying the strengths of rodent and cat studies with the translation power offered by non-human primates is a potential strategy for advancing knowledge gained from animal models.

## Insights on neuropathology

**Figure 3:**
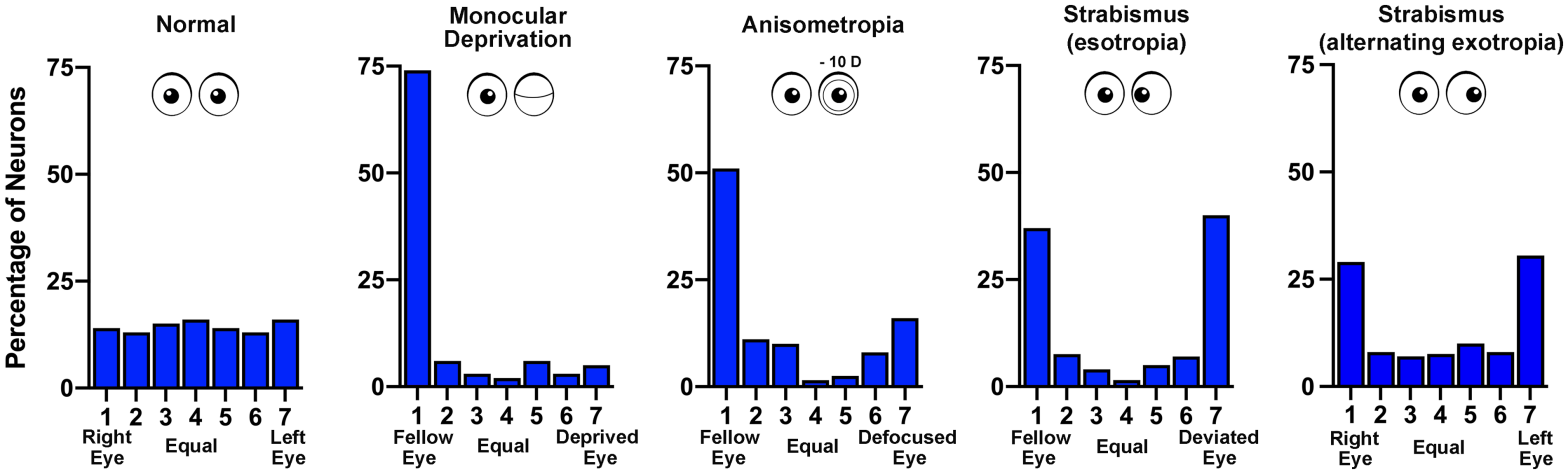
Ocular dominance histograms compare the effects of different rearing conditions that produce amblyopia. The ocular dominance of neurons sampled from the visual cortex of monkeys was measured by assessing the responsivity to stimulation of either the left or right eye. In normal animals there is a similar number of neurons connected to the right (group 1) and left (group 7) eye, with neurons connected to both eyes either equally (group 4) or somewhere in between (groups 2, 3, 5, 6; data from Kiorpes et al., 1998). Following monocular deprivation there is a strong shift in ocular dominance so that most neurons respond only to the non-deprived (fellow) eye (data from Hubel et al., 1977). Anisometropia produced by rearing with a contact lens placed in one eye also causes a shift in ocular dominance away from the affected eye, and like other amblyopia subtypes, reduces cortical binocularity (data from Kiorpes et al., 1998). Strabismus (esotropia or exotropia) does not result in disconnection of the affected eye but rather reduces the number of neurons responsive to both eyes – binocular cells (esotropia data from Kiorpes et al., 1998; exotropia data from Economides et al., 2021).

We are more than half a century removed from the seminal discoveries of Hubel and Wiesel (Wiesel and Hubel, 1963a,b) and much progress has been made. Their work spawned a vast collection of studies delineating innumerable effects of early visual deprivation. Yet still, the underlying neural pathology that gives rise to the functional consequences of MD is not fully understood. In cats, monkeys and mice, early MD precipitates a weakening and loss of excitatory synaptic connections serving the deprived eye (Thorpe and Blakemore, 1975; Hubel et al., 1977; Shatz and Stryker, 1978; Antonini and Stryker, 1993; Antonini et al., 1999; Coleman et al., 2010). This is a presumed consequence of binocular competition, in part because comparable changes are not observed in the region of V1 receiving input only from the contralateral eye (the monocular segment). The MD-induced redistribution of excitatory terminals produces a shift in cortical ocular dominance that leaves the deprived eye with fewer and weaker synaptic connections (Tieman, 1984). This alteration in connectivity results in a loss of cortical responsiveness to stimulation of the deprived eye (Wiesel and Hubel, 1963a; LeVay et al., 1980; Frenkel and Bear, 2004; Figure 3). Therefore, reduction in the number and strength of excitatory connections is considered the basis of MD-induced amblyopia (Dews and Wiesel, 1970; Giffin and Mitchell, 1978). A similar but less severe shift in cortical ocular dominance is observed in monkeys made anisometropic during the critical period (Kozma and Kiorpes, 2003; Rittenhouse et al., 2006). That these two types of amblyogenic rearing, form deprivation and anisometropia, result in a marked shift in ocular dominance away from the affected eye (Figure 3) suggests a proportionate loss or weakening of excitatory synapses as the primary cause of visual impairment (Khibnik et al., 2010). The loss of input decreases neuronal spatial sampling density, the so-called *spatial undersampling* hypothesis (Levi and Klein, 1986; Wang et al., 1998).

In contrast to the effect of MD, strabismic monkeys (esotropic or exotropic) do not exhibit a loss of synaptic connections serving the affected eye in V1. Both eyes are about equally connected to the visual cortex, although there is a precipitous decrease in the percentage of neurons receiving input from both eyes (Kiper and Kiorpes, 1994; Economides et al., 2021). This loss of binocularity in V1 is a feature shared across all amblyopia subtypes (Figure 3) and can be explained by a uniform set of assumptions for how excitatory synapses modify after MD and strabismus (see, e.g., Clothiaux et al., 1991). However, observations in V1 leave unexplained how strabismus degrades visual perception in only one eye to cause unilateral amblyopia. The prevailing view is that inputs serving the deviated eye are submerged by intracortical inhibition, an adaption to avoid double vision (Sireteanu, 1982; reviewed in Sengpiel and Blakemore, 1996). Chronic interocular suppression may play an active role in the development and progression of amblyopia in all subtypes. Binocular suppression of (and by) the impaired eye is not specific to strabismic amblyopia. There is evidence for persistence of interocular suppression within the visual cortex of monkeys made amblyopic by experimentally induced anisometropia (Hallum et al., 2017), as well as in humans with deprivation amblyopia (Hamm et al., 2017). Although the origin of neural suppression is not currently known, evidence from strabismic and anisometropic monkeys indicates that the capacity for suppression from both the amblyopic and fellow eyes is intact (Hallum et al., 2017; Economides et al., 2021). In other words, while the obstruction of normal binocular vision stimulates a redistribution of thalamocortical and binocular horizontal excitatory connections (Antonini and Stryker, 1993; Tychsen and Burkhalter, 1995; Trachtenberg and Stryker, 2001), inhibitory connections appear to be preserved and are not appreciably different from normal (Smith et al., 1997; Hallum et al., 2017; Economides et al., 2021). It will be important to determine if this result is observed after MD.

## Therapeutic innovations

The many factors that frustrate traditional treatments for amblyopia underscore the need to advance the standard of care. The emergence of binocular experience-based therapies is an attempt to address the shortcomings of traditional treatment. Dichoptic display therapy, for instance, aims to reduce suppression by the fellow eye and promote binocular vision by displaying a stronger stimulus to the weaker eye. Movies or video game stimuli have been used to promote patient engagement and treatment compliance. These and similar therapies have produced a promising degree of visual recovery in children and adults with anisometropic and/or strabismic amblyopia (Hess et al., 2010; Li et al., 2010; Vedamurthy et al., 2015; Holmes et al., 2016; Žiak et al., 2017; Birch et al., 2019b; Xiao et al., 2022). However, an efficacy review by The American Academy of Ophthalmology concluded that there was no evidence to support the use of binocular treatment as a substitute for standard patching or penalization for the common forms of amblyopia (Pineles et al., 2020). Further, binocular treatment has had mixed results when administered to children with deprivation amblyopia: children with MD from unilateral cataract showed modest or no improvement in visual acuity or contrast sensitivity following contrast-balanced binocular treatment (Hamm et al., 2018; Birch et al., 2020). Children with dense congenital cataracts consistently showed no improvement even when cataracts were removed at 4–6 weeks after birth (Birch et al., 2020). Additional emerging treatments for amblyopia include short-term patching of the amblyopic eye (inverse occlusion) that can promote durable recovery (Lunghi et al., 2018; Zhou et al., 2019), and also behavioral training therapy that can improve visual acuity in children and adults (Vedamurthy et al., 2015; Pineles et al., 2020; Kadhum et al., 2023). To our knowledge, the efficacy of these therapies has yet to be tested in patients with deprivation amblyopia.

Over the past decade, a bevy of novel approaches have been used to correct the effects of MD in animals. Several of these have elicited recovery in rodents by selective targeting of specific brain molecules or processes that regulate neural plasticity (Maya Vetencourt et al., 2008; Morishita et al., 2010; Silingardi et al., 2010; Grieco et al., 2020; Venturino et al., 2021). Other manipulations have produced recovery or have elevated plasticity potential through experiential manipulations such as environmental enrichment or exposure to 60 Hz light flicker, which appear to work by modulating GABAergic inhibition (Sale et al., 2007; Greifzu et al., 2014; Venturino et al., 2021). Complete elimination of visually-driven activity through brief dark exposure can also enhance plasticity and promote recovery from MD following reintroduction of the animals to a lighted environment (He et al., 2007). The motivation for using dark exposure derives from studies, both theoretical and experimental, that have shown the threshold for Hebbian synaptic strengthening is changed by periods of reduced activity in the visual system (Bienenstock et al., 1982; Kirkwood et al., 1996; Cooper and Bear, 2012). Once removed from darkness, visually-driven impulses promote strengthening of weak synapses serving the amblyopic eye (reviewed in Leet et al., 2022). The mechanism for this effect appears to include the modification of NMDA receptor structure and function (Quinlan et al., 1999; Philpot et al., 2001), as well as reconfiguration of the extracellular matrix surrounding thalamocortical synapses and inhibitory neurons (Murase et al., 2017). The beneficial effect of dark exposure on plasticity and recovery has now been demonstrated in three species across multiple labs: mice (Erchova et al., 2017); rats (He et al., 2007; Montey and Quinlan, 2011); and cats (Duffy and Mitchell, 2013; Gotou et al., 2021). However, dark treatment for human amblyopia is impeded by the logistical demands required to implement its clinical application. Dark therapy has also failed to promote recovery at older ages in cats (Duffy et al., 2018; Holman et al., 2018).

**Figure 4:**
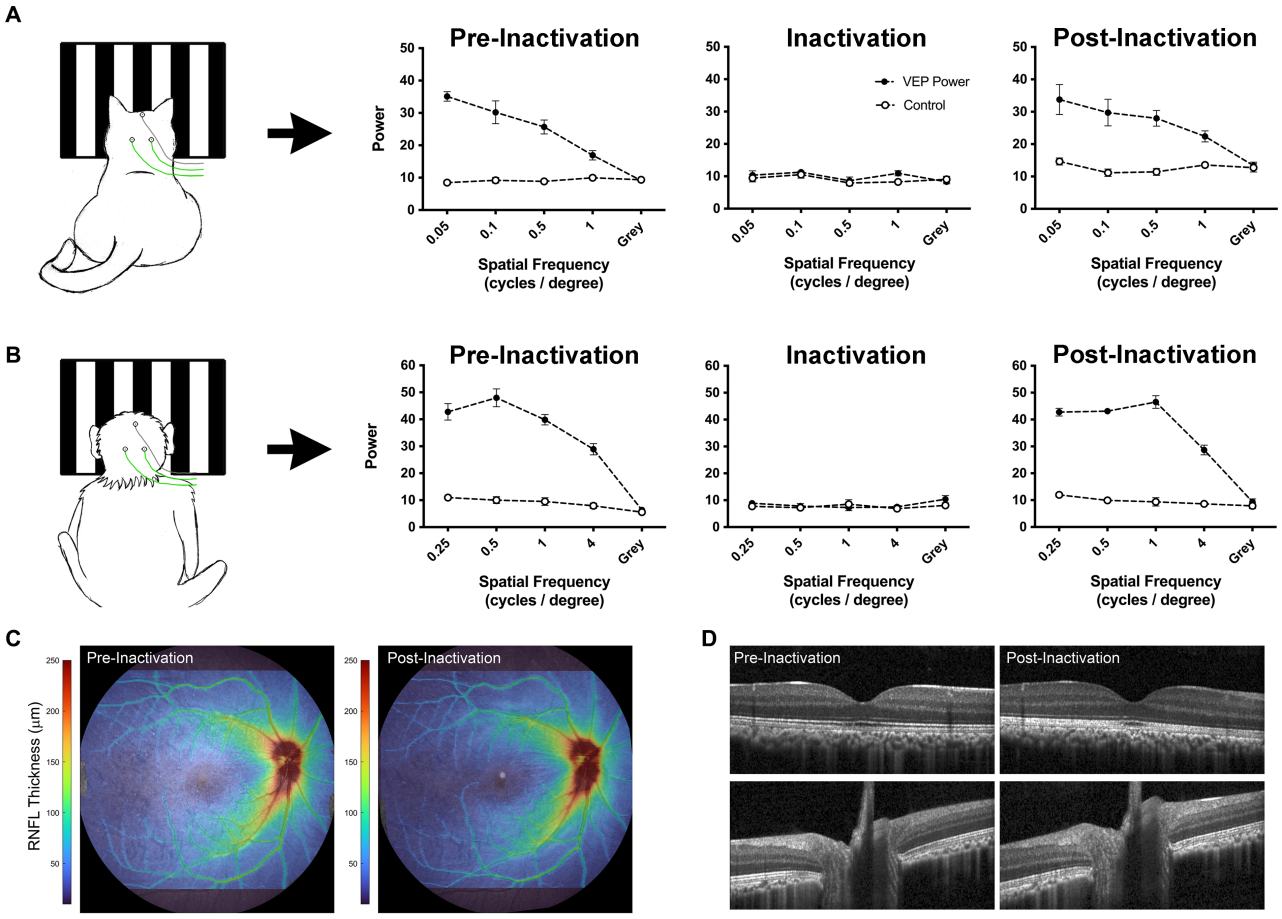
Data from cat and monkey revealing that the effect of intravitreal injection of TTX is reversible. VEPs (solid circles) measured from V1 in a cat (**A**; Duffy et al., 2023) and monkey (**B**; preliminary data) using scalp electrodes show a reduction to non-visual baseline levels (open circles) after TTX injection. Measurement of VEPs post-inactivation reveal a full recovery back to pre-inactivation levels for both species, indicating that the effect of inactivation on VEPs is temporary. OCT scans acquired from the monkey displayed in panel **(B)** demonstrate comparable retinal nerve fiber layer (RNFL) thickness between pre- and post-inactivation measurements **(C)**. Similarly, individual registered b-scans suggest no change in retinal or optic nerve anatomy following injection **(D)**. Monkey VEPs were collected for an experiment in which 4 TTX injections were delivered into the right eye over 4 weeks (one injection per week). Pre-inactivation VEPs were measured from right V1 at 8 months of age. Inactivation VEPs were measured 24 h after the first injection. Post-inactivation VEPs were taken 1 week after the final of four injections. OCT scans were acquired using the Spectralis OCT system (Heidelberg Engineering, Heidelberg, Germany), after pupils were dilated with 1% tropicamide. Scans acquired included a high resolution 55×45 degree raster scan, and 20×20 degree high speed raster scans centered on the optic nerve head and macula. Images were processed using neural network-based segmentation algorithms previously described (Srinivasan et al., 2022).

An alternative approach to reduce cortical activity is silencing retinal ganglion cells by intraocular injection of tetrodotoxin (TTX). A single intravitreal microinjection of TTX, a potent voltage-gated sodium channel blocker, can eliminate retinal output activity for approximately 48 h (Stryker and Harris, 1986; Linden et al., 2009; Fong et al., 2016). In comparison to occlusion therapy that eliminates only visually-driven activity, inactivation of the retina eliminates both visually-driven and spontaneous activity. In the case of MD, only dominant eye inactivation is required to markedly attenuate visual cortex activity because the amblyopic eye is incapable of driving normal cortical activity due to its weak connections. Freed from suppression by the dominant eye during the period of inactivation, the deprived eye’s excitatory synapses can recover via long-term synaptic potentiation (Clothiaux et al., 1991; Fong et al., 2021; Leet et al., 2022). Empirical evidence supporting this theoretical framework comes from cat studies (Kratz et al., 1976; Smith, 1981) as well as human case reports (Klaeger-Manzanell et al., 1994; El Mallah et al., 2000; Vagge et al., 2020; Resnick et al., 2023) that demonstrate post-critical period recovery from MD after loss or damage to the fellow eye. Recent studies have leveraged this knowledge to investigate retinal inactivation as a treatment for amblyopia caused by MD. Inactivation of the dominant eye in MD mouse or cat produces recovery of visually-evoked potentials (VEPs) when applied *after* the classical critical period (Fong et al., 2021; Hogan et al., 2023). Anatomical recovery also occurs. Neurons post-synaptic to the MD eye grow to normal size (Duffy et al., 2018). To be considered as a treatment for human MD, it will be of paramount importance to demonstrate full recovery of the inactivated eye in a primate model. Assessments to date in cats and monkeys have revealed no ocular pathology after inactivation for up to 10 days (Foeller and Tychsen, 2019; DiCostanzo et al., 2020; Duffy et al., 2023; Hogan et al., 2023). Figure 4 demonstrates restoration of VEPs following brief monocular inactivation in a cat (A) and macaque monkey (B). In both species, VEPs measured after ~10 days of inactivation were restored to pre-inactivation levels about 1 week after the final TTX injection. Assessment of the inactivated monkey eye using optical coherence tomography (OCT) revealed no retinal nerve fiber or ganglion cell layer abnormalities after inactivation (Figures 4C,D). Future studies are aimed at determining if retinal inactivation can enable recovery from deprivation amblyopia in monkeys, as well as assess the influence of age. Success in the primate model could pave the way to human studies.

## Conclusion

Amblyopia caused by MD is a rare and debilitating visual impairment that responds poorly to conventional therapy. Novel treatments being developed in human patients largely exclude those with MD because it is so resistant to therapy. Animal models offer a unique opportunity to address this unmet need. Investigation and development of unconventional amblyopia therapies cannot easily be pioneered in humans. With regard to inactivation therapy, the next logical step is to investigate its efficacy in primates. If successful, this innovation in MD treatment could be extended to target all types of amblyopia.

## Data availability statement

The original contributions presented in the study are included in the article/supplementary materials, further inquiries can be directed to the corresponding author.

## Ethics statement

The animal study was approved by University Committee on Laboratory Animals at Dalhousie University, and the Institutional Animal Care and Use Committee at the University of Houston. The study was conducted in accordance with the local legislation and institutional requirements.

## Author contributions

All authors listed have made a substantial, direct, and intellectual contribution to the work and approved it for publication.
